# Union of the Medical Societies of London

**Published:** 1906-11-17

**Authors:** 


### The Hospital

A Journal of

The Medical Sciences and Hospital Administration.

Vol. XLI.—No. 1,052. SATURDAY, NOVEMBER 17, 1906.

### Union of the Medical Societies of London.

Upon previous occasions we have expressed our cordial sympathy with the proposal to establish a union of the several Metropolitan medical societies. The present state of affairs is satisfactory neither from a professional nor a financial standpoint. It involves much overlapping and duplication of the machinery necessary to the organisation and working of the societies, and in this way money, time, and effort are alike wasted. Further, the separate existence and isolation of a number of professional societies means a great deal of repeated, and therefore needless, discussion, and many feeble and poorly attended meetings. More important still, it involves a divorce of the more specialised lines of professional work from the main currents of medicine and surgery, and such separation is bad in both directions. It means, on the one hand, a risk of narrow views and ill-informed practice; and on the other, the absence from societies charged with broad interests of those able to contribute special and peculiar information, which, though of local origin, may have an intimate bearing on general questions. In short, the existing separation opposes rather than facilitates the growth of a sound and broad medical doctrine and practice, and emphasises divisions which are not in harmony with the natural history and complexion of the processes of disease.

It is well known that the committee charged with the task of producing a scheme of amalgamation has found that to provide universal satisfaction was impossible. In affairs of this kind, whilst the voices of the critic and the discontented are loudly heard, it seems to be no one's business to express gratitude or appreciation. So far as our own judgment goes, we believe that, altogether apart from the fate awaiting the committee's proposals, the profession owes a debt of gratitude to those who in discharging a laborious task can have had no other motive than that provided by a lofty and single-minded public spirit.

Unfortunately, it must now be recognised that the proposal for the amalgamation of the societies is in danger of collapse. The committee having considered the various criticisms and comments passed on their provisional scheme have issued amended proposals, which certainly go far to meet suggested difficulties. These allow more complete self-government to the several sections of the proposed new society and also secure an adequate representation of each section both on the central council and the editorial committee. To meet the claims advanced on behalf of members of individual sections, which under the scheme will correspond to existing societies, the committee make very liberal concessions. They suggest that such members shall receive a printed copy, issued monthly, of the transactions not only of the section to which they happen to belong, but of all the sections; or, alternatively, it shall be open to any member to have an annual copy of the proceedings of the section in which he is interested. Upon the question of the admission of women the committee has exercised similarly a wise and skilful judgment. Women may not at the outset become Fellows of the new society, and this, whether we approve or disapprove, must be recognised as in harmony with general professional opinion. But care is to be taken that nothing in the constitution of the society shall prevent the election of women to the Fellowship in the future, if it should seem good to the society to take this step; and, further, all women who are at present members of societies taking part in the amalgamation scheme may become members of the corresponding sections in the new society.

The committee's proposals, amended as just stated, and with certain minor modifications, have now been sent to the societies interested, and it rests with each of these to decide for or against union on the lines suggested. Necessarily, therefore, there is considerable discussion in progress. Whilst some are stirred by the appeal for fusion in a larger unity, others stand for the preservation of independence; but, on the whole, it seemed, until recently, not unlikely that the former would carry the day. The opponents of union were perhaps the more demonstrative, but that there existed a steady body of conviction in favour of union was beyond question. The general position was indeed well illustrated by the experience of one of the existing societies, the council of which sent out postcards asking for the opinions of the members and at the same time expressing their own opposition to union; the result, doubtless to the surprise of the council, showed a decided majority in favour of amalgamation. This is provided by the final decision of the Medical Society, one of the most important and influential of existing societies, not to take part in the scheme—a decision which will probably exercise a determining influence upon many of the other societies and upon the whole scheme. Whether the amalgamation committee can or can not give further help we cannot say. But most earnestly would we counsel, on the part of all interested, considerate and serious speech and action, for wanting these, the prospect of union must almost certainly disappear for the present generation.

